# Coexistence of two sympatric predators in a transitional ecosystem under constraining environmental conditions: a perspective from space and habitat use

**DOI:** 10.1186/s40462-023-00421-1

**Published:** 2023-10-02

**Authors:** Chloé Warret Rodrigues, James D. Roth

**Affiliations:** Department of Biological Sciences, University of Manitoba, 50 Sifton Road, Winnipeg, MB R3T 2N2 Canada

**Keywords:** Arctic tundra, Exploitation competition, Interference, Intraguild interactions, Range expansion, Species coexistence

## Abstract

**Background:**

Range expansion of species, a major consequence of climate changes, may alter communities substantially due to competition between expanding and native species.

**Methods:**

We first quantified size differences between an expanding habitat generalist, the red fox (*Vulpes vulpes*), and a circumpolar habitat specialist, the Arctic foxes (*Vulpes lagopus*), at the edge of the Arctic, where climate-related changes occur rapidly, to predict the likelihood of the larger competitor escalating interference to intraguild killing. We then used satellite telemetry to evaluate competition in a heterogeneous landscape by examining space use early during the foxes' reproductive period, when resource scarcity, increased-food requirements and spatial constraints likely exacerbate the potential for interference. We used time-LoCoH to quantify space and habitat use, and Minta's index to quantify spatio-temporal interactions between neighbors.

**Results:**

Our morphometric comparison involving 236 foxes found that the potential for escalated interference between these species was high due to intermediate size difference. However, our results from 17 collared foxes suggested that expanding and native competitors may coexist when expanding species occur at low densities. Low home-range overlap between neighbors suggested territoriality and substantial exploitation competition for space. No obvious differential use of areas shared by heterospecific neighbors suggested low interference. If anything, intraspecific competition between red foxes may be stronger than interspecific competition. Red and Arctic foxes used habitat differentially, with near-exclusive use of forest patches by red foxes and marine habitats by Arctic foxes.

**Conclusion:**

Heterogeneous landscapes may relax interspecific competition between expanding and native species, allowing exclusive use of some resources. Furthermore, the scarcity of habitats favored by expanding species may emphasize intraspecific competition between newcomers over interspecific competition, thus creating the potential for self-limitation of expanding populations. Dominant expanding competitors may benefit from interference, but usually lack adaptations to abiotic conditions at their expansion front, favoring rear-edge subordinate species in exploitation competition. However, due to ongoing climate change, systems are usually not at equilibrium. A spread of habitats and resources favorable to expanding species may promote higher densities of antagonistically dominant newcomers, which may lead to extirpation of native species.

**Supplementary Information:**

The online version contains supplementary material available at 10.1186/s40462-023-00421-1.

## Introduction

Interspecific competition is a structuring force of carnivore communities [1, 2]. Wherever ecologically similar species are sympatric, competition can occur over limited shared resources with varied intensity levels [3, 4], depending on the similarity of the competitors' ecological niches [5, 6]. Two forms of competition are classically described (e.g., [3]). Exploitation competition is indirect, as competitors negatively affect each other by depleting a resource; its strength thus depends on spatio-temporal patterns of resource abundance [7]. Interference competition is direct, as individuals prevent others from accessing resources along a behavioral continuum from passive blocking to interspecific killing (e.g. [8]); its strength thus increases with competitor density and may not depend on resource availability (e.g. [7, 8]). Exploitation and interference competition typically increase together [9], likely because the rate of encounter of consumers with both competitors and their prey are driven by movement patterns [10].

Niche theory forecasts niche optima separation as a condition for similar species to coexist [6, 11]. Hence, although interspecific competition is a frequent community feature, it seldom leads to competitive exclusion, indicating coexistence mechanisms must be widespread [4, 12]. Examples of coexistence mechanisms include (1) differential tolerance to abiotic factors, which can lead to spatial interdependence between the population growth rate and a given resource density [13], (2) temporal or spatial segregation to avoid interference (e.g. [14]), (3) habitat or food-resource partitioning to allow exclusive use of resources [15, 16] — and thus the persistence of a given density of each competitor based on those resource dynamics. Niche partitioning is, therefore, contingent on heterogeneity along the niche axes [13].

Furthermore, when species have similar body sizes, and overlap widely in their use of resources, the likelihood of interference encounter increases [1]. The likelihood of interspecific killing, however, depends non-linearly on the magnitude of body size difference, being maximal at intermediate (i.e., 41.4–88.3%) size differences [17]. Carnivore interactions are usually asymmetric, and the likelihood of interference is highest at intermediate body-size differences, because at large differences dietary overlap is usually reduced, and at small differences the risk of injury or death from interference encounters is too high, even for the dominant competitor [17]. The subordinate species may thus shift their realized niche in response to a dominant competitor [6]. Empirical examples included generalist subordinate species that altered their resource selection [18, 19], habitat or space use [20], or activity peaks [21].

Arctic warming and other anthropogenic influences have permitted boreal forest species to expand their range onto the Arctic tundra, where they may compete with native species [22–24]. In particular, harsh winter conditions historically prevented red foxes (*Vulpes vulpes*) from becoming established on the tundra, but during the twentieth century, their northern range limit expanded > 1700 km towards the North pole [24]. The presence of red foxes within the distribution of Arctic foxes (*Vulpes lagopus*), which range throughout the Arctic, may thus elicit competitive interactions between the two foxes, given their ecological similarities. Both species are central-place foragers [25, 26], specifically when they reproduce, because they depend on dens for shelter and to protect their offspring from predators [19]. In North America, these two species feed preferentially on arvicoline rodents (lemmings and voles) year-round [27], but are also opportunists and use alternative resources if the preferred prey is less available or if an alternative prey becomes particularly abundant [28, 29]. Geese (*Branta canadensis* and *Anser caerulescens*), for example, provide an abundant source of food for tundra predators throughout summer [30], and in winter, the sea ice may provide foxes with alternative resources to terrestrial prey [27, 28].

We examined the potential for competition and segregation between red foxes and Arctic foxes in and near Wapusk National Park, in northern Manitoba, Canada, where red foxes recently became established on the coastal Arctic tundra between tree line and Hudson Bay and now reproduce in sympatry with Arctic foxes [31, 32]. Both species use the same dens and share tundra prey [31, 33]. Red foxes evolved as a boreal-forest species [34], and thus have poor adaptation to the harsh abiotic conditions of the tundra, where they usually occur in low density or discontinuously, and may resort to drastic behavioral adjustments to cope with environmental harshness [24, 32, 35]. We focused this study on the most critical period for the foxes, between the beginning of gestation and pup emergence (mid-March to mid-June). Foxes may compete for pre-existing dens when they start reproducing, as the ground is still frozen and they cannot excavate new ones [31]. Food resources slowly increase throughout this period, but geese only start arriving during the first week of May [36], and the median hatch date of Canada goose eggs occurs during the 4^th^ week of June [37]. Competition over food resources is, thus, likely the strongest during this period, because of increased energetic needs due to gestation and lactation, while resources are still scarce, and foxes are spatially constrained to remain near breeding dens that shelter vulnerable pups. However, the landscape in transitional areas is by essence heterogeneous, which may help relax competition between ecologically similar species. For example, red foxes commonly avoid the sea ice, unlike Arctic foxes, which use this habitat to compensate for terrestrial food shortage [28, 32]. The sea ice may thus offer an opportunity for resource partitioning between the two species.

We first assessed size differences between red and Arctic foxes to predict the likely magnitude of interference risk for the Arctic foxes and, thus, to what extent Arctic foxes should avoid red foxes (see Additional file 1: Appendix A). We hypothesized that the potential for exploitation competition during this critical period is substantial. However, habitat heterogeneity offers opportunities for the two species to partition resources, thus relaxing a high potential for a red-fox-dominated interference competition driven by intermediate body size difference (sensu [17]; Additional file 1: Appendix A). We consequently predicted that during this time period, (P1) red foxes have larger home ranges than Arctic foxes, reflecting their difference in body size, (P2) resource scarcity exacerbates competition, so little home-range overlap occurs [38], (P3) risks of interference induce asymmetric spatiotemporal use of the shared area between heterospecific neighbors, in favor of the red fox, and (P4) red and Arctic foxes partition habitat and time-use of shared areas.

## Methods

### Study area

**Fig. 1:**
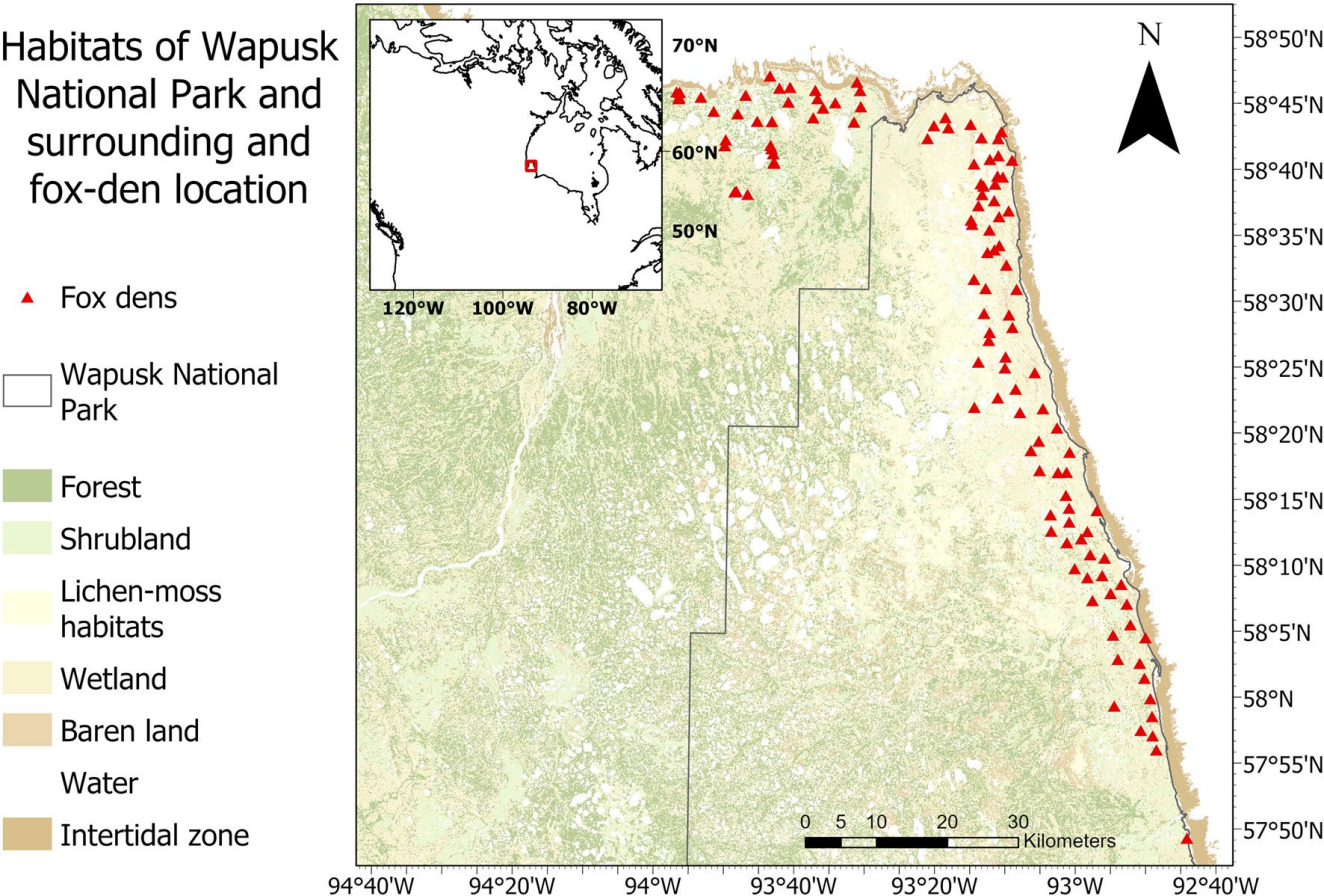
Habitat map of the study area in Wapusk National Park and its surrounding area [43]

We studied the spatiotemporal interactions of red and Arctic foxes in Wapusk National Park and the Churchill Wildlife Management Area (58°N, 94°W) (Fig. 1). This area is part of the Hudson Bay Lowlands, a uniformly flat (< 200 m elevation) wetland bordering the south-western shore of Hudson Bay to the western shore of James Bay [39]. Hudson Bay exerts a strong cooling effect on the area. Onshore winds from the Bay dominate during most of the growing season, and are an important factor limiting tree growth [40, 41]. The interchanging influence of the offshore and onshore winds favors abrupt and sometimes important changes in air temperature [40]. Thus, three biomes merge in this area: the tundra and marine ecosystem of Hudson Bay to the north and east, and the boreal forest to the south and west. We defined the low tide line as the start of the sea ice [42], i.e., beyond the intertidal flats (see Fig. 1). The proximity of the boreal forest to our study area may favor a continuous source of red foxes, which are likely to increase on the tundra as winters become milder. The presence of transitional habitat, with patches of trees increasing in density and size near the boreal forest and along the river corridors [42], may offer additional opportunities for red foxes to spatially segregate from Arctic foxes. Furthermore, both fox species are harvested in the area, providing the opportunity for morphometric comparisons of foxes collected from local fur trappers to assess the potential for interference based on body size differences (Additional file 1: Appendix A).

### Capture and satellite telemetry

Between 2017 and 2019 we captured 10 red foxes and 13 Arctic foxes using padded leghold traps (Softcatch # 1.5, Oneida Victor Ltd, USA) and Tomahawk live traps (Model CB12DD-36, Tomahawk Live Trap Co., WI). Traps were deployed each year between March and May opportunistically on tundra dens and spruce islets, wherever we identified signs of fox activity (see [32] for additional details on trapping procedures). We targeted areas with more signs of fox activity to capture neighbors. However, because it is virtually impossible to collar all neighbors from a given area (e.g., foxes transiting, leaving, or settling after we left), we captured small groups of neighbors scattered throughout the whole study area. Average distance between neighbors in April varies between 5.4 and 7.7 km [31], and we know the locations of all dens in the area where we trap [31, 44]. We can, therefore, ensure that no other fox lives between the neighbors we caught. We fitted all captured foxes, which were easily handled without chemical restraint, with an Iridium satellite collar (#4170 or 4270, Telonics, Mesa, Arizona, USA; ~ 100 g, or 2–4% of body mass) before releasing them at the site of capture. Median handling time was 25 min. [11–50] from our arrival at the trap station to fox release. All handling procedures were approved by the University of Manitoba Animal Care Committee (Protocol F17-012). Our research was carried under Parks Canada permits WAP-2017–25781 and WAP-2018-27938, and Manitoba Wildlife Scientific Permits WB20226 and WB21856.

### Movement analyses

Between March 15 and June 15, our satellite collars collected one location per 1.5 to 2 h (12–16 daily locations). We first plotted all fox tracks in ArcGIS 10.3 (ESRI [45], Redland, CA, USA) to identify movement strategies: residency and dispersal [32]. Foxes that obviously dispersed were excluded from subsequent analyses. To confirm range residency, we next produced empirical variograms (i.e., relocation semi-variance, which measures variability in the distance between location pairs plotted against time lags between relocations) of foxes deemed residents, using package ctmm 0.6.0 [46]. Convergence toward an asymptote suggests range residency. One case was ambiguous, but a test for range shift using package marcher v0.0.2 [47] suggested that he shifted his center of activity. We, thus, excluded the short period before the range shift [48].

Excursions are conceptually different and distinct from movements exhibited within the home range and may differ in length and frequency between individuals. We, thus, removed excursions to improve homogeneity among foxes to estimate their home ranges [49]. To identify excursions, we first produced a density plot of the distribution of distances between locations and the track centroid (using ArcGIS 10.3), and identified outliers using a one-sided Hampel filter with R packages fitdistrplus 1.1.1 [50] and rcompanion 2.3.25 [51], where:

1 $${\text{upper bound }} = {\text{ median }}\left( {{\text{Tukey}} - {\text{transformed distance}}} \right) \, + {\text{ 3 median absolute deviations }}\left[ {{32}} \right].$$

We produced home-range estimates with Time Local Convex Hulls (T-LoCoH; [52]), a family of non-parametric methods to build Utilization Distributions (UD) that extends the classic LoCoH non-parametric methods [53] by integrating both time and space in the construction of the local hulls associated with each location. The Time Scaled Distance metric (TSD) transforms the time interval between locations into a distance on a third axis of the Euclidian space, by scaling the individual’s maximum theoretical velocity (i.e., the maximum observed velocity between two consecutive points: *v*_max_) with a dimensionless scaling factor(*s*). If *s* = 0, time is ignored (hulls are space selected). As *s* increases, points that are far away in time get pushed apart regardless of their proximity in space: hulls become time and space selected (see [52] for a detailed explanation). Given the median position autocorrelation of our tracks of 9.4 h, and our 12 to 16 locations per day, we defined a 12-h period of interest for all foxes. We selected individual-specific values of *s* based on the recommendations and tools provided in the package T-LoCoH v.1.40.07 [52]. Our *s* values ranged between *s* = 0.01 and *s* = 0.1. To create the hullsets, we defined the Number of Nearest Neighbors (NNN) using the adaptive method (i.e., nearest neighbors are all points whose cumulative distance to the focal point is ≤ *a*), which is less sensitive to outlying locations and better suited when location densities are heterogeneous [53]. We selected an *a*-value for each animal using the recommendations and graph tools provided in the T-LoCoH package to minimize the risks of type I (excluding used areas) and type II (including unused areas) errors.

We first aggregated and sorted the hulls based on the number of enclosed nearest neighbors. The resulting isopleths thus represent the likelihood of occurrence, and these home range estimates can be used to assess the intensity of use. We used the 95% (home range) and 50% (core area) isopleths to compare space requirements between red and Arctic foxes (P1) and assess the potential for exploitation competition (P2).

A key property of the T-LoCoH is that hulls include points spatially close but temporally distant [52]. These points indicate recurring visits to the hull, based on a specified temporal threshold (the inter-visit gap, i.e., in our case 12 h) that defines the time to pass before another observation counts as a new visit. They, thus, contain time-use information from which we can derive time-use metrics. We used this property to produced behavioral maps to assess the potential for spatio-temporal and resource segregation between the two fox species (P4). We sorted the hulls based on the Number of Separate Visits (NSV)—a measure of revisitation rate—and the Mean Number of Locations per Visit (MNLV)—a proxy for duration of use — based on our 12-h period of interest as the inter-visit gap. To be conservative in estimating the key resources for our foxes, without excluding important resources that may represent a potential for segregation, we selected the 50% isopleths to minimize both type I and type II errors.

We measured the extent of NNN home-range overlap between heterospecific and homospecific neighbors (P2 and P3). Foxes were considered neighbors when the distance between their home range boundaries (based on the 95% isopleths NNN estimate) was less than the radius of a red fox's home range (i.e., < 3.2 km; see Results). We calculated home-range overlap of a dyad (i.e., 2 neighboring foxes regardless of species) as:

2 $${\text{Overlap }} = \, \left[ {\left( {{\text{area}}_{{{\text{AB}}}} /{\text{home range}}_{{\text{A}}} } \right)*\left( {{\text{area}}_{{{\text{AB}}}} /{\text{home range}}_{{\text{B}}} } \right)} \right]^{{0.{5}}}$$

where area_AB_ is the area delimited by the overlap of the two home ranges and home range_A_ and home range_B_ are the individual home range areas of individuals A and B.

We then quantified the spatial and temporal use of the shared areas with package wildlifeDI v. 0.4.1 [54] using Minta’s [55] set of coefficients (LA:Ā, L_B:B̄_, and L_ixn_) and compared them for each type of dyad (heterospecific, homospecific red foxes and homospecific Arctic foxes). The spatial coefficients, L_A:Ā_ and L_B:B̄_, indicate the probability of finding A and B in a specific zone given the proportion of areal overlap [55]. The spatial behavior towards the shared area can thus be characterized as random (L_A:Ā_ or L_B:B̄_ ~ 0), attraction (L_A:Ā_ or L_B:B̄_ > 0), or avoidance (L_A:Ā_ or L_B:B̄_ < 0). Based on the response of each animal, a dyad’s spatial response to the shared area can be symmetric (same response), asymmetric (opposite response), or singular (only one individual shows a significant response at *p* < 0.05). The L_ixn_ coefficient is calculated from the ratio of simultaneous presence and absence to solitary presence in the shared area [54, 55]: when L_ixn_ ~ 0, individual’s temporal use is random, whereas L_ixn_ > 0 indicates simultaneous use of the shared area, and L_ixn_ < 0, a solitary use of the shared area [54, 55]. We defined simultaneous locations using a 5-min. buffer related to variation in location calculation by the collars. We used these indices to further indicate the possibility of interference competition between the fox species (P3) and used the L_ixn_ in the context of symmetric attraction for the shared area as evidence of time segregation (P4).

To compare key habitats for red *versus* Arctic foxes, we reclassified the Canadian Landcover 2015 vegetation map [43] and the intertidal zone (a key habitat that was missing from the Canadian Landcover map) of the Wapusk National Map Ecotype map [42]. We obtained the following relevant categories: barren land, wetland, tundra, shrubland, forest, intertidal flats, and sea ice (see Additional file 1: Table S1). We then exported the NSV and MNLV hulls created in T-LoCoH as shapefiles in ArcGIS Pro 2.4 (ESRI, 2020), which we intersected with the reclassified habitat map. We calculated the proportion of each habitat type, excluding 2 Parks Canada compounds and water (ponds, lakes, and streams).

### Statistical analyses

We conducted all analyses in R version 4.0.5 [56] using R Studio version 1.4.1717 [57]. We checked all our models for: residual normality and applied transformations when necessary, heteroscedasticity and adapted our tests accordingly, and presence of outliers that we reviewed individually to keep or discard [58].

We compared species’ requirements for space (P1) using a GLMM (family Gaussian, link identity) from package lme4 v.1.1–25 [59] and lmerTest v.3.1.3 [60], controlling for fox-pair ID as a random effect. Two dyads of red foxes had a home range overlap of at least 85%, while the median of all overlaps was 6%: 1 dyad was a mated pair, and the other was 2 females who may have been mother-daughter, based on the age difference suggested by tooth wear. We also considered them as a pair to avoid pseudoreplication. To compare home-range overlap within and between species (P2), excluding overlaps between foxes that belonged to a pair, we used a two-sided permutation test (t statistic, n_perm_ = 999). We repeated the analysis for the overlap between core areas using Wilcoxon signed-rank tests. We assessed the possibility that interference (P3) and time segregation (P4) occurred: we tested if the behavioral response (random, attraction, or avoidance) and the symmetry of that response differed between the type of fox neighbors (heterospecific, Arctic- and red-homospecific pairs) with a Fisher exact test. We tested if a fox was more likely to avoid a heterospecific neighbor than a homospecific one using a GLMM, using the odds of solitary use as the response variable and the type of neighbor as the explanatory variable (family Gaussian and identity link), controlling for fox ID as a random effect, and allowing the variance to differ between neighbor types [61, 62] with the varIdent function of package nlme v.3.1.152 [63]. Finally, to assess the potential for resource segregation, we first compared the habitat composition of the NSV- and MNLV-range areas of the two species using MANOVA (e.g. [64]). Habitat variables from both NSV and MNLV datasets had multivariate normal distribution. Variance of the NSV dataset was homogenous so we report results from the R manova function, which uses Pillai’s Trace test statistic. We dealt with heteroscedasticity in the MNLV dataset using a parametric bootstrap resampling method (n_iter_ = 10,000) with package MANOVA.RM v.0.5.2 [65], and report the modified ANOVA-type statistic (MATS; [66]). To control for pseudo-replication, we used the median value from replicated individuals and from individuals in a pair.

## Results

**Fig. 2:**
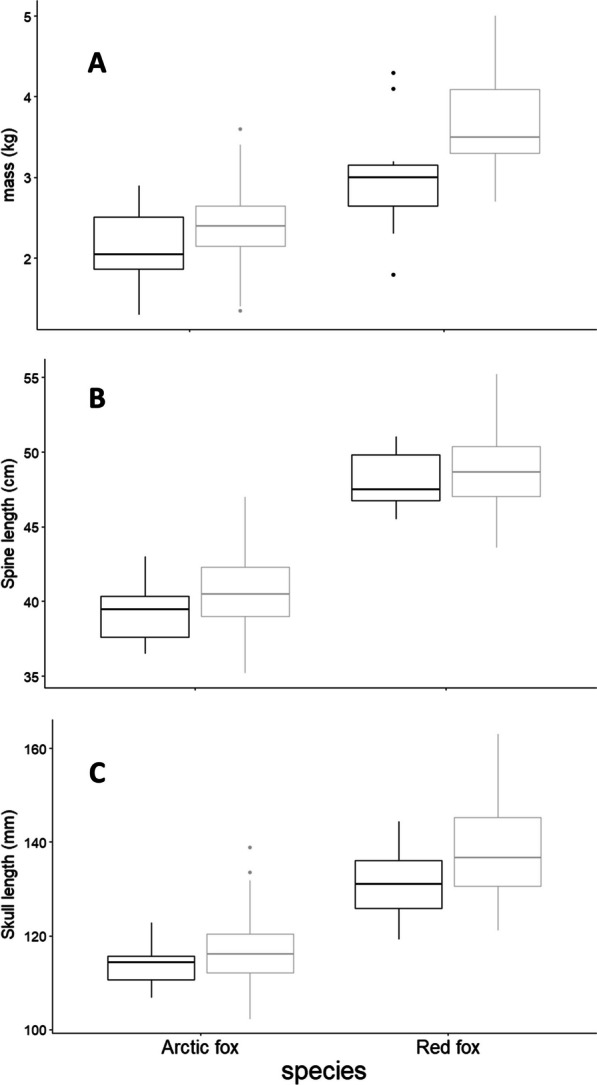
Body measurements of Arctic and red fox carcasses (females in black, males in grey) collected by local fur trappers near Churchill, Manitoba, Canada. **A** mass, **B** spine length, and **C** skull length

Our morphometric comparison of 236 fox carcasses (n_red male_ = 33, n_red female_ = 10; n_Arctic male_ = 175, n_Arctic female_ = 18) collected from local fur trappers suggested the potential for interference between red and Arctic foxes is high, red foxes being larger and heavier than Arctic foxes with intermediate size difference (Fig. 2; Additional file 1: Appendix A).

### Fox capture and observations

Between March 15 and June 15 of 2017 to 2020, we tracked 17 foxes that exhibited range residency (Additional file 1: Fig. S2), and 4 that did not. The resident foxes — 8 red and 9 Arctic foxes — yielded a total of 12,840 locations after removing their excursions [48]. Because 2 red foxes were present for 2 seasons and 1 red fox for 4 seasons, we obtained 22 home ranges over the 4 years (13 red fox and 9 Arctic fox).

### Space requirements and potential for exploitation competition

**Fig. 3:**
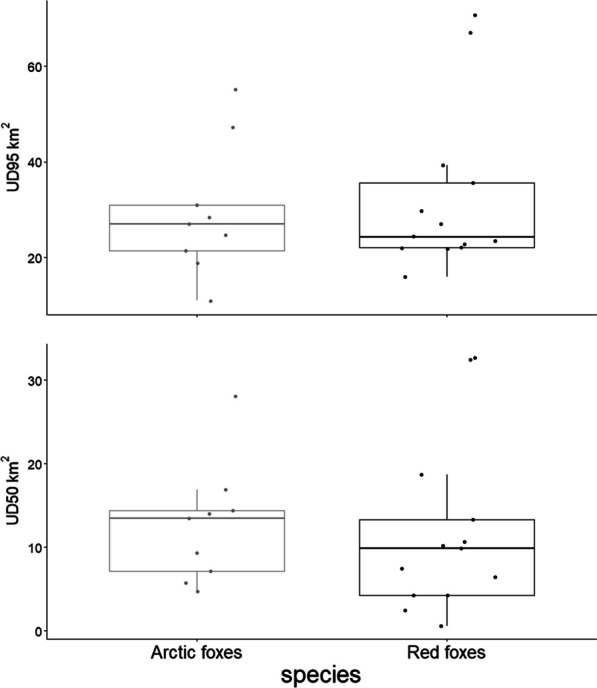
Home range area of Arctic and red foxes (March–June) in northeastern Manitoba, Canada. Home ranges were estimated using Time Local Convex Hulls (*a*-method; Lyons et al. 2013)

Arctic and red foxes had home ranges and core areas of similar size over the period of interest despite their difference in body size (GLMM: home range: t_13.16_ = 0.24, P = 0.82, n_red_ = 13, n_Arctic_ = 9; core area: t_13.20_ = -0.57, P = 0.58, n_red_ = 13, n_Arctic_ = 9; Fig. 3; Additional file 1: Appendix A). The overlap of NNN-home ranges between neighboring foxes was generally low (Table 1), and the overlap of core areas virtually nonexistent (except in one case where 2 male Arctic foxes neighbors overlapped highly with an index of 0.28, representing 0.25 and 0.31 of their core areas). The amount of home-range or core area overlap was similar between heterospecific and homospecific neighbors (permutation *t*-test: *t* = 0.814, *P* = 0.352; Wilcoxon signed-rank test: Z = − 0.93, *P* = 0.56; n_hetero_ = 3, n_homo_ = 12), and within each type of homospecific neighbors (*t* = − 0.156, *P* = 0.95; Z = − 0.51, *P* = 0.68; n_red-red_ = 8, n_Arctic-Arctic_ = 4).

**Table 1:** Overlap index of the NNN-home range (95% UD) and NNN-core area (50% UD) of red foxes (RF) and Arctic foxes (AF) fitted with a satellite collar in northern Manitoba, Canada, between 2017 and 2020. Overlap summary statistics are displayed for homospecific (AF-AF and RF-RF) and heterospecific (AF-RF) neighbors (*NNN* Number of Nearest Neighbors)

UD*	Species pair	Mean	SE	Min	Max	Median	*n*
95	AF-AF	0.05	0.04	0.00	0.16	0.01	4
	RF-AF	0.08	0.04	0.02	0.16	0.06	3
	RF-RF	0.05	0.02	0.00	0.15	0.05	8
50	AF-AF	0.07	0.07	0.00	0.28	0.00	4
	RF-AF	0.00	0.00	0.00	0.00	0.00	3
	RF-RF	0.00	0.00	0.00	0.00	0.00	8

^*^Utilization distribution

### Interference and segregation

**Fig. 4:**
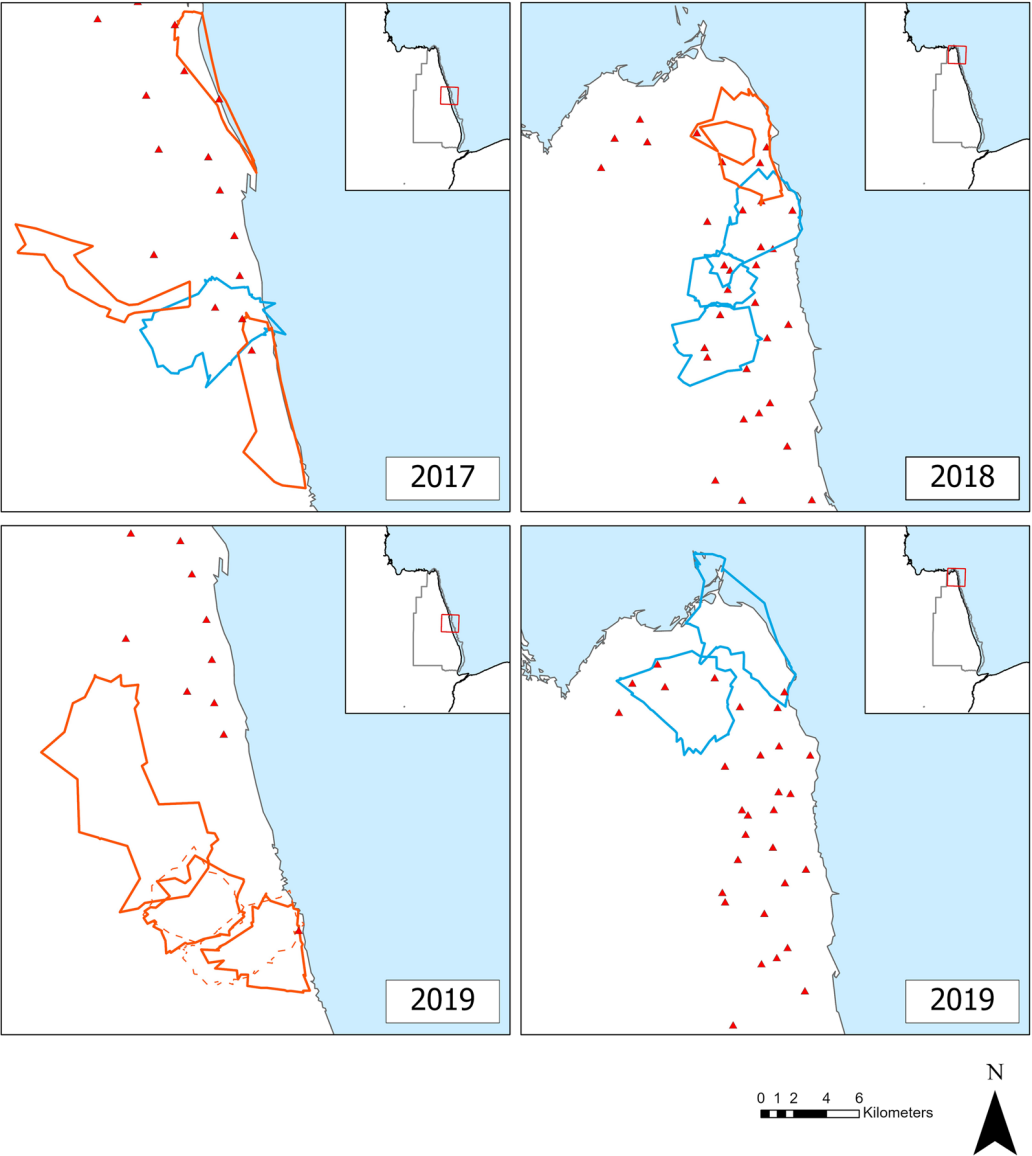
Overlapping fox dyads over the study period (March-June) in northeastern Manitoba, Canada. Arctic foxes are in blue, red foxes in orange (for clarity we used dashed lines when foxes were living together, and their home ranges overlapped substantially). Dens are shown as red triangles

Twelve of the 15 dyads shared some area of their home range, including all 3 heterospecific dyads (Table 2; Fig. 4). The 3 heterospecific dyads showed different spatial and temporal responses. In 1 dyad, both foxes used their shared area randomly. In one case, the Arctic foxes was attracted to the shared area, and the odds of the two foxes using the area together were nearly 6 times higher than expected despite the spatial indifference of the female red fox to the shared area. In the last heterospecific dyad, the Arctic foxes showed spatial avoidance. Two dyads of Arctic foxes showed singular attraction to their shared area, and their odds of solitary use ranged from 2 to 5 times higher than expected (Table 2). The last dyad of Arctic foxes, involving 2 males sharing a large area (of both their 95% and 50% UD), showed a strong symmetric attraction to that shared area and the L_ixn_ = 1.49 with *p* < 0.001 indicated strong temporal attraction. Their odds of simultaneous use were 33 times higher than expected, and both males used the shared area solitarily in a similar way (pair Gi-Gh, Table 2). These results suggest simultaneous temporal use and symmetrical-spatial attraction. The spatio-temporal relationships between the 6 red fox dyads suggested dominance relationships in half cases, with asymmetric attraction to the shared area, and one fox having much higher odds of solitary use than expected while the other had much lower odds of solitary use than expected. The remaining red fox dyads showed singular attraction to the shared area or symmetric attraction but solitary use. The spatial response to a shared area did not differ between dyad types (Table 2; *P* = 0.15). On a temporal axis, however, the odds of solitary use of a shared area were higher for homospecific than heterospecific neighbors (GLMM: t_9_ = 3.78, *P* = 0.004, n_hetero_ = 6, n_homo_ = 18).

**Table 2:** Minta’s indices and cells’ odds [55] for each overlapping fox dyad using data from foxes collared in northern Manitoba, Canada, between 2017 and 2020

Dyad type*	Fox ID		LA:Ā	PA:Ā	L_B:B̄_	P_B:B̄_	L_ixn_	P_ixn_	Dyad's response		Odds**			
	A	B							Spatial	Temporal	$\frac{nAB}{{pABn}}$	$\frac{nA0}{{pA0n}}$	$\frac{n0B}{{p0Bn}}$	$\frac{n00}{{p00n}}$
RF-AF	AB	MM	− 0.09	0.52	0.45	0.15	− 0.10	0.86	Random	Random	1.15	1.42	0.82	0.90
	FJ	MM	0.45	0.54	− 1.83	0.02	− 0.55	0.88	Singular avoidance	Solitary trend	0	0.14	1.34	0.85
	W	Gh	0.17	0.92	0.76	0.00	1.21	0.00	Singular attraction	Simultaneous	5.62	1.21	0.70	0.80
AF-AF	Gi	Gh	2.35	0.00	2.29	0.00	1.49	0.00	Symmetric attraction	Simultaneous	33	3.66	3.83	0.34
	Gi	GK	0.79	0.15	1.78	0.00	− 2.11	0.53	Singular attraction	Solitary trend	0	4.99	1.88	0.83
	DL	T	1.17	0.16	1.60	0.01	− 2.11	0.85	Singular attraction	Solitary trend	0	4.00	2.61	0.80
RF-RF	A	S	0.88	0.00	− 0.50	0.00	− 0.45	0.30	Asymmetric	Solitary trend	0.84	0.55	2.17	0.89
	A	Br	2.13	0.00	− 0.42	0.00	− 0.70	0.00	Asymmetric	Solitary	2.7	0.53	6.42	0.74
	S	LR	1.68	0.00	0.07	0.84	0.66	0.01	Singular attraction	Simultaneous	7.91	0.61	3.85	0.73
	S	I	1.33	0.00	− 0.47	0.12	− 1.51	0.18	Singular Attraction	Solitary trend	0	0.55	3.16	0.82
	LR	Br	1.15	0.00	1.75	0.00	− 2.32	0.01	Symmetric attraction	Solitary	0	4.40	2.84	0.71
	I	Br	− 1.01	0.10	0.72	0.03	− 0.90	0.67	Asymmetric	Solitary trend	0	1.90	0.34	0.92

^*^AF Arctic fox, *RF* Red fox

^**^Odds are ratio of frequency of observed (*n*) to expected (*p*) simultaneous use (AB), solitary use (A0 and B0), and non-use (00) of the area of overlap shared by individuals A and B [55]. Together with the Minta's index, they reflect the probability of attraction to or avoidance of the area of overlap. L_A:Ā_ and L_B:B̄_: probability of finding A and B in home range_A_, home range_B_, and area_AB_ given the proportion of areal overlap. L_ixn_: ratio of simultaneous presence and absence to solitary presence in the shared area

**Fig. 5:**
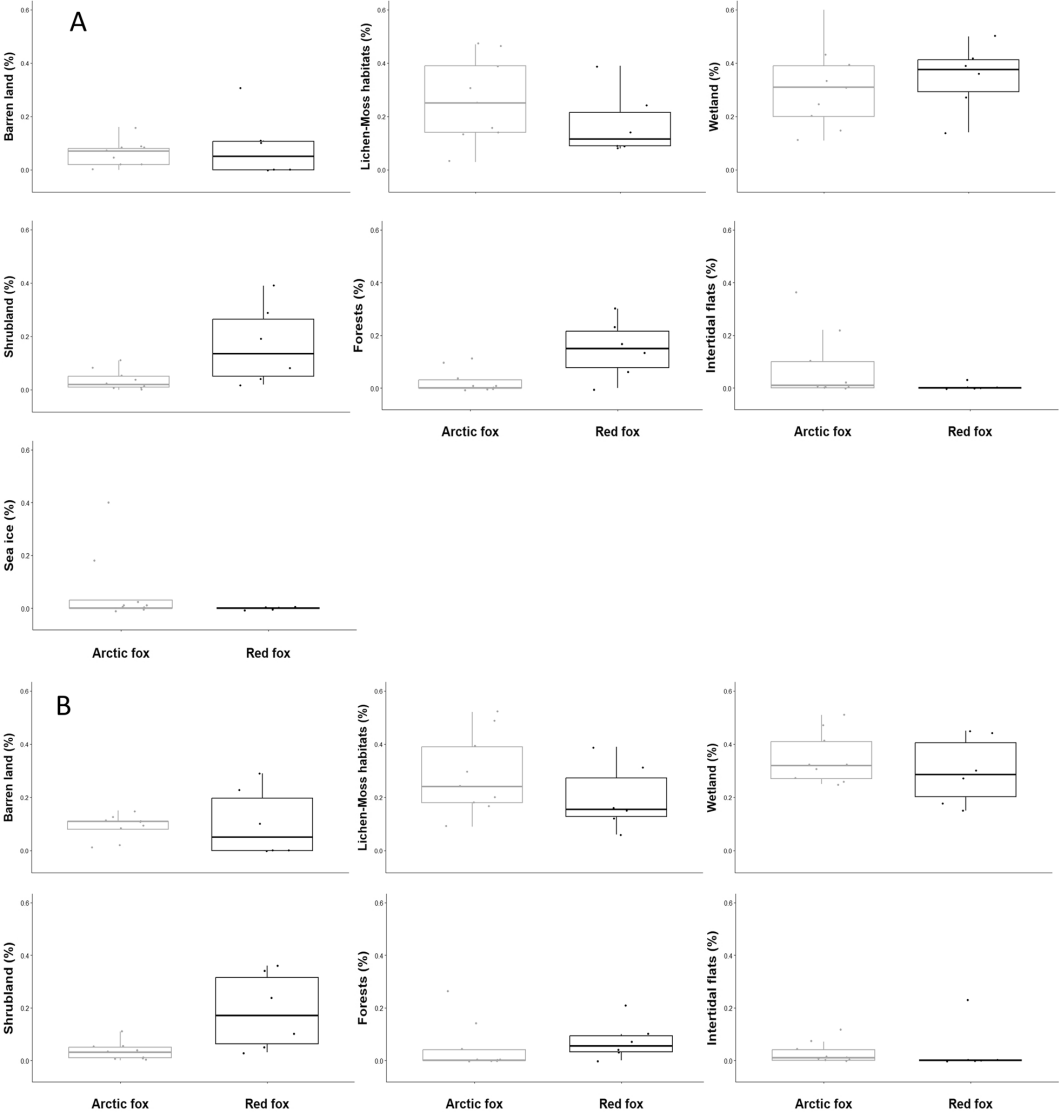
Comparison of each habitat proportion constituting the 50% UD MNLV **A** and NSV **B** ranging areas for red and Arctic foxes in Northern Manitoba

Habitat composition of both the MNLV (MANOVA: MATS = 3.50, *P* = 0.045) and NSV (MANOVA: F_6,8_ = 3.89, Pillai = 0.74, *P* = 0.040) ranges differed between species (Fig. 5; Additional file 1: Table S2). Red foxes never used the sea ice and only one individual used a relatively large area of intertidal flats in his NSV and MNLV ranges (accounting for 23% and 3% respectively), whereas no Arctic foxes used denser forested habitats (i.e., sub-polar broadleaf, needleleaf and mixed forests; see Additional file 1: Tables S1 and S2). Sea ice was never part of the Arctic foxes’ NSV home ranges but composed 3 to 39% of the MNLV home ranges of 3 out of 9 Arctic foxes. Only one red fox used mixed forest habitat, but this type of forest is scarce in Wapusk National Park. Two red foxes had established their home ranges on the coast, in pure tundra habitat, and thus did not include any patch of denser forest; one did not even have access to the sparse-canopy forest (i.e., subpolar taiga needleleaf forest; Table S1). Conversely, the 4 other red foxes’ MNLV home ranges were composed of 1 to 12% of denser forest patches and 5 to 22% of the sparse-canopy forest type (see Additional file 1: Tables S1 and S2).

## Discussion

Overall, we did not find evidence of conspicuous interference, nor a strong asymmetry in interactions in favor of the red fox. Yet, Arctic and red foxes belong to the same genus, with an intermediate difference in mass, spine length and skull length (ratios of red to Arctic foxes metrics were 1.4, 1.2 and 1.2, respectively), which is usually associated with unbalanced co-existence and strong interference [17]. Evidence of partitioning between these two species was scarce. Should resources be limiting, our results, therefore, suggest that strong exploitation competition could occur between red and Arctic foxes over the niche axes we tested but the patchiness of the landscape holds the potential to facilitate the coexistence of the two species.

Based on body-size differences and red foxes’ lack of physiological adaptation to food scarcity and extreme tundra conditions [67, 68], we expected red foxes to have maintained home ranges at least 40% larger than Arctic foxes [69]. Instead, red foxes ranged similarly to Arctic foxes, and to the rest of the winter (November 1–May 15; [32]) despite geese becoming available late May. Arctic foxes, however, ranged more than they typically do during winter [32], or in the High Arctic [35]. Arctic foxes have evolved physiological adaptations to prey scarcity and low temperatures [67, 68] but may have higher energetic costs due to reproduction than red foxes. Indeed, Arctic foxes have the highest litter weight (controlled for gestation time and compared to female weight) of all canids, including red foxes [70], and the largest litters among carnivores, with a mean of 10 pups in Canada [71], whereas red foxes have a maximum of 5 to 7 pups [72, 73]. In addition, March to mid-May, is the reproductive period of ringed seals (*Phoca hispida*) [74]. From pup birth to weaning, ringed seals use birth lairs on the landfast ice, near the coast [75, 76], and Arctic foxes hunt and scavenge on seal pups [77]. During the target period of this study, Arctic foxes may commute to the sea ice more extensively to use this resource, thus increasing the size of their home ranges.

The virtual absence of overlap between neighbors given the scarcity of prey suggests that both species behaved territorially as evidenced elsewhere (e.g., [78, 79]). High territoriality is consistent with the hypothesis of scarce, scattered, and unpredictable resources [38], which suggests that geese do not become an important resource until later (i.e., after foxes start to reproduce) and that conditions at the beginning of the reproductive period are challenging regarding resource acquisition.

No overlapping fox dyad symmetrically avoided their shared area, despite resource scarcity. When neighbors shared space, the overlap and level of spatial attraction for the shared area were similar for homospecific and heterospecific dyads. The competitive exclusion principle states that for two species to coexist, intraspecific competition should be stronger than interspecific competition [13, 80]. Therefore, both species using space similarly and spatially avoiding neighbors regardless of species suggests that exploitation competition for space between expanding and native species could be substantial if their densities increased or resources became scarcer. In such case, the spatial axis would, thus, offer little possibility for expanding and native species to coexist since it did not favor intraspecific over interspecific competition [13].

On a temporal scale, however, intraspecific avoidance tended to be greater than interspecific avoidance. Particularly, we only found evidence of dominant-subordinate interactions between red foxes (i.e., asymmetric spatial attraction and temporal pattern of use with Minta’s index). The stress caused by challenging abiotic conditions, emphasized by increased energetic needs due to reproduction, may negatively affect the competitive abilities of leading-edge populations. Stronger intraspecific competition in expanding species could induce self-limitation, which may benefit native species, especially as generalist species continue to expand (e.g., because of climate changes) because it could locally maintain a certain density threshold of expanding species, at which weaker native species could persist [3, 8, 81].

The usage patterns of sea ice, intertidal flats, and forests differed markedly between the two species in terms of intensity (i.e., as designated by the MNLV ranges) and frequency (i.e., NSV range). Some Arctic foxes used the intertidal flats relatively often and travelled sometimes far from their dens to remain on the sea ice for long periods, suggesting that these individuals foraged there, potentially detecting marine mammal carcasses from their terrestrial range [82]. In contrast, red foxes scarcely used the intertidal flats and never used the sea ice (despite the coastal home ranges of 5 out of 8 individuals). Thus, the marine environment may help the two species to coexist, providing (nearly) exclusive resources to the Arctic fox. Furthermore, only red foxes used the forest patches, both intensively and frequently, suggesting these habitats are important, and may notably be used as hunting grounds [83]. These results are consistent with Churchill fox diet [33, 84]. Both fox species in the study area relied on tundra prey (rodents and geese), but each species used different alternative prey [33]. Arctic foxes consumed marine items, suggesting their greater use of the tidal flats was to detect carcasses on sea ice [82]. Red foxes heavily relied on snowshoe hares (*Lepus americanus*) [33], suggesting that forest patches around Churchill offer alternative prey to tundra rodents, such as snowshoe hares, red squirrels (*Tamiasciurus hudsonicus*) and red-backed voles (*Myodes gapperi*), which they may use to cope with the scarcity of tundra prey. These forest patches increase in density along a south-western gradient; they also occur along the river corridors, specifically towards the southern parts of Wapusk and on slightly warmer sites [42]; red foxes could also favor these forested patches because they offer shelter from the wind, thereby lessening the abiotic challenges imposed by tundra conditions. Alternatively, the different habitat composition may simply reflect a southeast-northwest gradient of red fox density that reflects the directionality of their range expansion. Increased habitat complexity may reduce competition and promote coexistence of similar predators, because they provide opportunity for exclusive resource use by each competitor [85].

The shape and size of home ranges depend on resource distribution and abundance, and on neighbors' space use. Space and habitat use within home ranges and incursions into neighboring territories are dynamic processes, likely adjusted (in time and space) according to movements of neighboring resident or transient competitors, whether they are conspecific or not [86]. It is virtually impossible to collar all individuals of a population, and effects of non-collared individuals on collared ones cannot be quantified. We, thus, only ever obtain a partial picture to infer the mechanisms driving movement patterns of collared individuals. Furthermore, the lifespan of satellite collars often comes at a trade-off with fix frequency; the coarser the resolution of the GPS fix schedule, the less likely real-time reaction to competitors at fine-time resolution. Although we did not detect strong patterns of avoidance between the two species, some finer-scale mechanisms, such as real-time tracking of competitors or reactions at short distances, may be at play [87]. For example, lynx and wolverine showed little spatio-temporal segregation at coarser scales, but active avoidance over short distances can be detected at finer scale, promoting co-existence between the two competitors [87].

Species interactions can range from facilitation to predation, and within the same context they are not necessarily mutually exclusive [88]. For example, individuals can engage in agonistic interactions resulting in intraguild predation [17] but also forage on each other’s kills (scavenging or kleptoparasitism). Coexistence is thus contingent on complex mechanisms, and interactions between two species vary depending on context (e.g., spatially, seasonally, along a gradient of abiotic conditions, prey abundance; [88]). Notably, prey abundance can be key in determining coexistence between predators that substantially overlap spatially, temporally and in their diet [88]. For example, interactions between red and Arctic foxes around Churchill included indifference and agonistic interactions where the pursuer was the smaller species. In the first case (Additional file 1: Fig. S3A), the heterospecific individuals were observed in town where they likely benefited from anthropogenic subsidies. In early June (second case Additional file 1: Fig. S3C), resources become more abundant because geese reproduce but pups are still vulnerable; the Arctic fox was, thus, likely defending its breeding den.

Some level of interference that we did not detect in the early reproductive season may occur during fall at least, as indicated by the infrequent but regularly observed intraguild killing events where red foxes dominated, as expected given their larger body size (Additional file 1: Fig. S3B). In fall, juveniles are dispersing, temperatures drop (likely increasing red fox energetic requirements), and resources become scarce, thus providing red foxes with a size advantage and strong motivation to escalate the aggression [17, 89]. However, high risks linked to interference may not always translate into spatial exclusion [89]. Dietary and behavioral flexibility are often particularly high in medium-size carnivores [90], and thus fine-scale spatiotemporal partitioning may be key in favoring larger-scale spatial coexistence, despite high risks of interference encounters (including interspecific killing; [89, 91]).

Interference during the reproductive period may be costly for both species, but these costs are not necessarily symmetrical. Theory predicts that interference must benefit (i.e., increase offspring production) the lesser exploiter to allow coexistence under the condition of an interference-exploitation trade-off [8]. Such a trade-off may apply to our fox populations: red foxes have the potential to benefit from interference and monopolize crucial resources, but Arctic foxes are likely better exploiters that may survive and reproduce under a larger range of resource conditions due to smaller size and higher physiological adaptation to cope with low temperature and food scarcity [6, 67, 68].

## Conclusion

Like in the Canadian High Arctic, red foxes around Churchill did not seem to exclude Arctic foxes from the vicinity of their home range by interference [35]. This observation differs markedly from those made in Eurasia or Alaska, where interference is strong—red foxes exclude Arctic foxes from breeding dens, and Arctic foxes spatially avoid red foxes [92–94]. In Eurasia and Alaska, red foxes are subsidized by anthropogenic resources, like human food waste [95], semi-domesticated reindeer, or roadkills [96, 97]. The absence of anthropogenic food sources in our study site and the Canadian High Arctic likely helped balance the competition between the fox species, because red foxes mostly rely on fluctuating prey, likely leading these populations to fluctuate in abundance [98]. The negative impact of red foxes on Arctic foxes increases with red fox abundance and decreases with variability of red fox abundance over time [98]. The absence of consistent food subsidies stabilizing red fox populations may, thus, be a major reason for the dramatically different outcome of red-Arctic foxes interactions between the Canadian Arctic and Fennoscandia or Alaska.

Coexistence is measured as a function of population trends in the long term [99]. When species are highly similar, competitive exclusion may even depend on stochastic processes [6], but the potential exists for local coexistence of generalist-expanding and native species, like it seems to be the case in our area given the current condition. However, most ecosystems are not at equilibrium, notably due to climate-related and anthropogenic changes. In the Churchill area, Arctic foxes are declining while red foxes remain stable [31, 100]. A higher relative abundance of red foxes may further accelerate an Arctic-fox decline linked to changes in environmental conditions (like the loss of sea ice), until red foxes become more common than Arctic foxes, or Arctic foxes become locally extinct.

Range expansion of species and competitive exclusion may be ongoing processes. Future changes may favor expanding species and compress the realized niche of their native competitors. In the Arctic, increased winter temperatures will likely lower the costs associated with thermoregulation for both tundra-native and expanding-boreal-forest species that currently occur as edge populations [39, 101, 102]. However, tundra species that rely on their ability to exploit alternative resources that will be negatively affected as the Arctic warms could lose the potential to exploit these alternative resources [100]. Native species highly adapted to their environment may experience range shifts towards areas where the conditions still allow them some exploitative advantages over poorly adapted expanding species, as abiotic conditions continue to change.

### Supplementary Information

**Additional file 1.** **Appendix A. Fig. S1.** Density histogram of body mass (pelt removed) of red foxes (in orange; n = 43) and Arctic foxes (in blue; n = 193) legally harvested in the Churchill area, Manitoba, Canada in 2017 and 2018. **Appendix B. Table S1.** Description of habitats selected from the Canadian Landcover 2015 vegetation map ("LC2015", adapted from Latifovic 2019) and reclassification into relevant habitats to fox activities to test for differences between species in the home range composition. **Table S2.** Proportion of each habitat type in the total area of each individual fox’s MNLV- and NSV-home range (50% UD) between March 15 and June 15 in northeastern Manitoba, Canada (habitat IDs described in Table S1). (MNLV: Mean Number of Locations per Visit; NSV: Number of Separate Visits). **Fig. S2.** Variograms of foxes identified as resident after inspection of their raw tracks at large time lags (25-40 days). AF = Arctic fox, RF = red fox. **Fig. S3.** Examples of interactions between Arctic and red foxes in the Churchill and Wapusk area in northeastern Manitoba, Canada. A) The two species can be observed tolerating each other, notably where they may access anthropogenic food subsidies (in town). B) Rarely but regularly, interference interactions can be lethal for the Arctic foxes. These extreme events were always observed in November (B. Debets, pers. obs., November 2014; J. Waterman, pers. obs., November 2017; D. Alcorn, pers. obs., November 2020), which, in Churchill, marks the beginning of food scarcity and harsher climate (Warret Rodrigues and Roth 2023). C) Arctic fox chasing a red fox from its den (interference interaction) in June, when geese have started reproduction and resources are becoming more abundant, but pups are likely born and highly vulnerable (both foxes remained alive at least until the camera stopped working the following week). Photos courtesy of Churchill resident Dave Allcorn (A; March 2022) and Dr. Jane Waterman (B; November 2017), and retrieved from our Reconyx trail camera by Sean Johnson-Bice (C; June 2021).

## Data Availability

Morphometry data are archived on Mendeley with the https://doi.org/10.17632/6bph26nct2.1. The dataset analyzed for the present study is available in the Movebank Data Repository: https://doi.org/10.5441/001/1.291 [48].
