# Supplementary material for: Coexistence of two sympatric predators in a transitional ecosystem under constraining environmental conditions: a perspective from space and habitat use
Source: Mov Ecol. 2023 Oct 2;11:60. doi: 10.1186/s40462-023-00421-1 (PMC10544556; doi:10.1186/s40462-023-00421-1)
Supplement: Supplementary file 1 — Additional file 1. Appendix A. Fig. S1. Density histogram of body mass (pelt removed) of red foxes (in orange; n = 43) and Arctic foxes (in blue; n = 193) legally harvested in the Churchill area, Manitoba, Canada in 2017 and 2018. Appendix B. Table S1. Description of habitats selected from the Canadian Landcover 2015 vegetation map ("LC2015", adapted from Latifovic 2019) and reclassification into relevant habitats to fox activities to test for differences between species in the home range composition. Table S2. Proportion of each habitat type in the total area of each individual fox’s MNLV- and NSV-home range (50% UD) between March 15 and June 15 in northeastern Manitoba, Canada (habitat IDs described in Table S1). (MNLV: Mean Number of Locations per Visit; NSV: Number of Separate Visits). Fig. S2. Variograms of foxes identified as resident after inspection of their raw tracks at large time lags (25-40 days). AF = Arctic fox, RF = red fox. Fig. S3. Examples of interactions between Arctic and red foxes in the Churchill and Wapusk area in northeastern Manitoba, Canada. A) The two species can be observed tolerating each other, notably where they may access anthropogenic food subsidies (in town). B) Rarely but regularly, interference interactions can be lethal for the Arctic foxes. These extreme events were always observed in November (B. Debets, pers. obs., November 2014; J. Waterman, pers. obs., November 2017; D. Alcorn, pers. obs., November 2020), which, in Churchill, marks the beginning of food scarcity and harsher climate (Warret Rodrigues and Roth 2023). C) Arctic fox chasing a red fox from its den (interference interaction) in June, when geese have started reproduction and resources are becoming more abundant, but pups are likely born and highly vulnerable (both foxes remained alive at least until the camera stopped working the following week). Photos courtesy of Churchill resident Dave Allcorn (A; March 2022) and Dr. Jane Waterman (B; November 2017), and retriev [file 40462_2023_421_MOESM1_ESM.docx]

# Appendix A. Morphometric comparison of Arctic foxes and red foxes near Churchill, MB, Canada

# Background

Red and Arctic foxes have similar requirements and use the same resources (food or dens). When ecologically similar species have similar body sizes, and overlap widely in their use of resources, the likelihood of interference encounter increases [1]. The likelihood of interference resulting in interspecific killing, however, depends non-linearly on the magnitude of body size difference, being maximal at intermediate size differences [2]. We assessed fox size differences to predict the likely magnitude of exploitation competition and interference risk for the Arctic fox and, thus, to what extent Arctic foxes should avoid red foxes.

# Methods

*Fox morphometrics. –* To predict the potential strength of interference competition, we quantified the size difference between the two fox species using carcasses of foxes legally harvested in our study area during the trapping season (November – March) [2]. We collected 236 fox carcasses  (n_red male_ = 33, n_red female_ = 10; n_Arctic male_ = 175, n_Arctic female_ = 18) from local fur trappers in Churchill after pelts were removed in 2017 and 2018, and kept them frozen until processing. We measured body mass using Pesola scales (5 kg and 10 kg), skull length from the rhinion to the occipital protuberance using electronic calipers, and spine length using a tape measure applied flat on the fox spine starting at the cervical C1 to the end of the sacrum. We extracted canines and X-rayed them using standard radiography techniques. We measured pulp cavity and tooth width at its widest point in ImageJ [3]. We then sent all teeth with a pulp cavity-tooth width ratio ≤40% (a threshold under which we considered animals were subadults [4]) to Matson’s lab (Manhattan, Montana, USA) for aging using cementum annuli count.

*Statistical analysis. –* We tested species difference in body length with a linear model using sex and species as predictors. To test species difference in body mass we used a generalized linear mixed model (GLMM: family Gaussian, link identity), controlling for age as a random effect, because age may affect foraging performance, and using the varIdent function to account for heteroscedasticity for the species variable [5]. We compared skull length (log-transformed) using a generalized least square model (GLS) with the varIdent function to allow different variance by species. We compared models with age, sex, species and their interaction terms because canids are sexually dimorphic (males are usually larger) and age can be strong driver of intraspecific variations in skull morphometry [6]. We selected the model with the lowest Akaike information criterion, with correction for small sample size [7], and only report the results from that model. The best models did not include interaction terms, nor age (possibly because of the large proportion of subadults in our samples). Both the GLMM and GLS were functions of the package nlme v.3.1.152 [8]. We then tested intraspecific sexual dimorphism with a series of linear models including only sex as a predictor. We tested these model residuals for outliers, deviation from normality, and homoscedasticity [9], and report no issue.

Donadio & Buskirk, [2] computed the proportional body size difference (BSD) between carnivores, with an arcsin square root transformation, as follows:

BSD = arcsin(SQRT((BM_L_ – BM_S_)/BM_L_)),

where BM_L_ is the body mass of the larger species and BM_S_ is the body mass of the smaller species. To assess how much the Arctic fox should avoid red foxes (because of interference-related risk of injury and mortality), we used their index to classify the body-size difference between species (i.e., small = [0%-41.3%], intermediate = [41.4%-88.3%], large = [88.4%-100%]), overall and for each sex combination. We conducted all these analyses in R version 4.0.5 (R Core Team, 2021) using R Studio version 1.4.1717 (RStudio Team, 2021).

# Results

We obtained 193 Arctic fox (190 subadults, 3 adults) and 43 red fox (32 subadults, 11 adults) carcasses from Churchill trappers that were not scavenged, with pelts removed. Red foxes had larger linear dimensions (Fig. 2), both in spine length (LM: t_233_ = 21.57, P < 0.001) and skull length (GLS: t_228_ = 13.00, P < 0.001, n_AF_ = 190, n_RF_ = 41). They were also heavier than Arctic foxes (GLMM: t_227_ = 10.49, P < 0.001; Fig. S1). In red foxes, males were heavier than females (t_41_ = 2.95, P = 0.005), but had similar linear dimensions (spine length: t_41_ = 0.99, P = 0.329; skull length: t_39_ = 1.49, P = 0.15). In Arctic foxes, males were heavier (t_191_ = 2.79, P = 0.006) and longer than females (spine length: t_191_ = 2.47, P = 0.014; skull length: t_188_ = 1.96, P = 0.052), although the evidence of sexual dimorphism in skull length was weaker. Red foxes and Arctic foxes had intermediate size difference, whether we compare both populations as a whole, or between species within each sex cohort. The overall BSD between red and Arctic foxes was 0.60. The BSD between male red foxes and male Arctic foxes was 0.62, between male red foxes and female Arctic foxes was 0.70, between female red foxes and male Arctic foxes was 0.45, and between female red foxes and female Arctic foxes was 0.50.

# Conclusion

Red foxes are larger and heavier than Arctic foxes with intermediate size difference, whether we compare both populations as a whole, or between species within each sex cohort. These results suggest the potential for interference between these species in our study area is maximal [2].

# References

1. Palomares F, Caro TM. Interspecific killing among mammalian carnivores. Am Nat. 1999;153:492–508.

2. Donadio E, Buskirk SW. Diet, morphology, and interspecific killing in carnivora. Am Nat. 2006;167:524–36.

3. Schneider CA, Rasband WS, Eliceiri KW. NIH Image to ImageJ: 25 years of image analysis. Nat Methods. 2012;9:671–5.

4. Grue H, Jensen B. Annual cementum structures in canine teeth in arctic foxes (*Alopex lagopus L.*) from Greenland and Denmark. Danish Rev Game Biol. 1976;10:1–12.

5. Zuur AF, Ieno EN, Walker NJ, Saveliev AA, Smith GM. Dealing with Heterogeneity. In: Zuur A, Ieno EN, Walker N, Saveliev AA, Smith GM, editors. Mix Eff Model Extensions Ecol with R. New York: Springer; 2009. p. 71–100.

6. Forbes-Harper JL, Crawford HM, Dundas SJ, Warburton NM, Adams PJ, Bateman PW, et al. Diet and bite force in red foxes: ontogenetic and sex differences in an invasive carnivore. J Zool. 2017;303:54–63.

7. Hurvich CM, Tsai CL. Regression and time series model selection in small samples. Biometrika. 1989;76:297–307.

8. Pinheiro J, Bates D, DebRoy S, Sarkar D, Team the RC. The nlme Package: Linear and Nonlinear Mixed Effects Models. R-project. 2007;

9. Zuur AF, Ieno EN, Elphick CS. A protocol for data exploration to avoid common statistical problems. Methods Ecol Evol. 2010;1:3–14.

# Figure


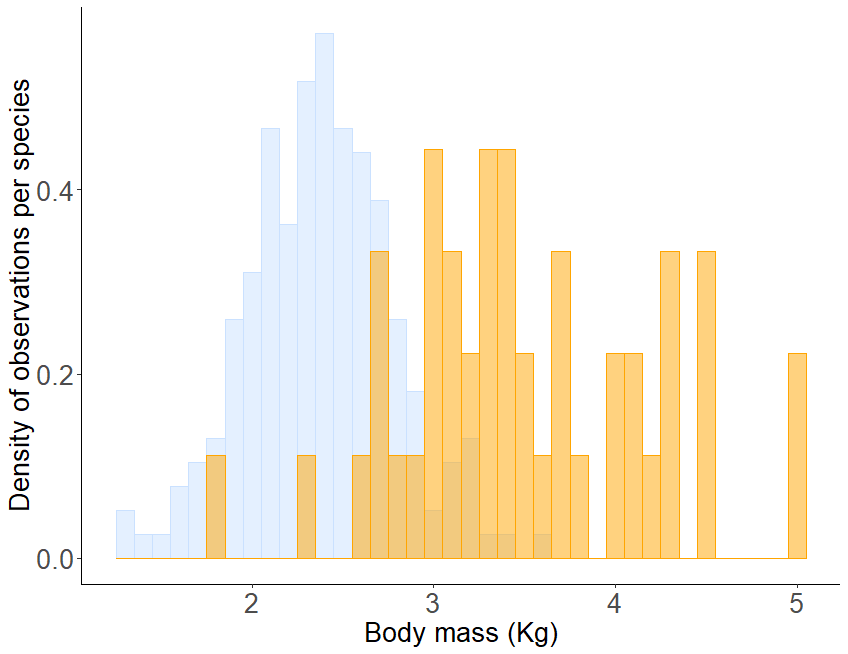


**Fig. S1.** Density histogram of body mass (pelt removed) of red foxes (in orange; n = 43) and Arctic foxes (in blue; n = 193) legally harvested in the Churchill area, Manitoba, Canada in 2017 and 2018.

# Appendix B. Additional supplementary information on resident Arctic fox and red fox space use during the reproductive period near Churchill, MB, Canada

**Table S1.** Description of habitats selected from the Canadian Landcover 2015 vegetation map ("LC2015", adapted from Latifovic 2019) and reclassification into relevant habitats to fox activities to test for differences between species in the home range composition

| **Habitat LC2015** | **Habitat reclassified** | **ID** | **description (LC2015)** |
| --- | --- | --- | --- |
| Barren land | Barren Land | 1 | Bare mineral material, vegetation accounts for <10% of total cover |
| Sub-polar barren-lichen-moss | Lichen-moss habitats | 2 | Mixture of bare area with lichen-moss accounting for at least 20% of total vegetation cover (typical of tundra) |
| Sub-polar shrubland-lichen-moss | Lichen-moss habitats | 2 | Dwarf shrubs with lichen and moss accounting for at least 20% of vegetation cover (typical of tundra) |
| Sub-polar grassland-lichen-moss | Lichen-moss habitats | 2 | Grassland with lichen and moss accounting for at least 20% of total vegetation cover (typical of tundra) |
| Wetland | Wetland | 3 | Herbaceous or woody vegetation influenced by water table at or near surface and present for a substantial part of the year |
| Sub-polar shrubland | Shrubland | 4 | Woody perennial plants <3m accounting for >20% of total vegetation cover |
| Sub-polar grassland | Shrubland | 4 | Herbaceous vegetation accounting for at least 80% of total vegetation cover interspersed between shrubland patches |
| Sub-polar needleleaf forest | Forest | 5 | Forest >3m accounting for >20% of total vegetation cover, needleleaf-tree species account for >75% of canopy coverage |
| Sub-polar taiga needleleaf forest | Forest | 5 | Woodlands, treed wetland with needleleaf-tree species >3m accounting for >5% of total vegetation cover, shrub and lichen understory often present, canopy variable and sparse |
| Sub-polar broadleaf deciduous forest | Forest | 5 | Forest >3m accounting for >20% of total vegetation cover, broadleaf deciduous species account for >75% of canopy coverage |
| Mixed forest | Forest | 5 | Forest >3m accounting for >20% of total vegetation cover, broadleaf and needleleaf tree species co-dominate, neither reaching 75% of total tree cover |
| Intertidal flats | Intertidal flats | 6 | Marine-terrestrial ecotone between high and low tides |
| Sea ice | Sea ice | 7 | Marine ecosystem of the Hudson Bay. Covered by ice during the study period |

**Table S2.** Proportion of each habitat type in the total area of each individual fox’s MNLV- and NSV-home range (50% UD) between March 15 and June 15 in northeastern Manitoba, Canada (habitat IDs described in Table S1). (MNLV: Mean Number of Locations per Visit; NSV: Number of Separate Visits).

|  |  |  |  |  | **Proportion** of habitat (ID)** | | | | | | |
| --- | --- | --- | --- | --- | --- | --- | --- | --- | --- | --- | --- |
| **species** | **year** | **Fox ID*** | **Estimator** | **area**** | **1** | **2** | **3** | **4** | **5** | **6** | **7** |
| **Arctic fox** | **2019** | B | MNLV | 23.25 | 0.07 | 0.13 | 0.20 | 0.02 | 0.03 | 0.10 | 0.39 |
|  |  |  | NSV | 18.08 | 0.11 | 0.24 | 0.32 | 0.03 | 0.04 | 0.07 | 0.00 |
| **Arctic fox** | **2019** | DL | MNLV | 11.89 | 0.16 | 0.16 | 0.15 | 0.04 | 0.00 | 0.22 | 0.03 |
|  |  |  | NSV | 17.56 | 0.15 | 0.17 | 0.25 | 0.04 | 0.00 | 0.12 | 0.00 |
| **Arctic fox** | **2018** | Gh | MNLV | 4.88 | 0.05 | 0.31 | 0.25 | 0.01 | 0.00 | 0.01 | 0.00 |
|  |  |  | NSV | 8.81 | 0.13 | 0.30 | 0.31 | 0.01 | 0.00 | 0.01 | 0.00 |
| **Arctic fox** | **2018** | Gi | MNLV | 8.51 | 0.09 | 0.39 | 0.31 | 0.01 | 0.00 | 0.00 | 0.00 |
|  |  |  | NSV | 5.98 | 0.11 | 0.49 | 0.26 | 0.01 | 0.00 | 0.00 | 0.00 |
| **Arctic fox** | **2018** | GK | MNLV | 4.60 | 0.08 | 0.46 | 0.39 | 0.01 | 0.00 | 0.00 | 0.00 |
|  |  |  | NSV | 7.96 | 0.09 | 0.52 | 0.32 | 0.00 | 0.00 | 0.00 | 0.00 |
| **Arctic fox** | **2019** | M | MNLV | 4.94 | 0.00 | 0.03 | 0.11 | 0.08 | 0.09 | 0.36 | 0.17 |
|  |  |  | NSV | 30.38 | 0.01 | 0.09 | 0.27 | 0.05 | 0.26 | 0.04 | 0.00 |
| **Arctic fox** | **2017** | MM | MNLV | 9.33 | 0.02 | 0.25 | 0.43 | 0.11 | 0.11 | 0.00 | 0.00 |
|  |  |  | NSV | 12.65 | 0.02 | 0.18 | 0.47 | 0.11 | 0.14 | 0.00 | 0.00 |
| **Arctic fox** | **2019** | T | MNLV | 13.74 | 0.02 | 0.14 | 0.60 | 0.00 | 0.00 | 0.02 | 0.00 |
|  |  |  | NSV | 9.86 | 0.11 | 0.20 | 0.51 | 0.01 | 0.00 | 0.01 | 0.00 |
| **Arctic fox** | **2017** | U | MNLV | 16.89 | 0.08 | 0.47 | 0.33 | 0.05 | 0.00 | 0.00 | 0.00 |
|  |  |  | NSV | 9.37 | 0.08 | 0.39 | 0.41 | 0.05 | 0.00 | 0.01 | 0.00 |
| **Red fox** | **2020** | A | MNLV | 21.73 | 0.10 | 0.08 | 0.27 | 0.19 | 0.30 | 0.00 | 0.00 |
|  |  |  | NSV | 33.54 | 0.10 | 0.12 | 0.27 | 0.34 | 0.03 | 0.00 | 0.00 |
| **Red fox** | **2017** | AB | MNLV | 3.83 | 0.00 | 0.24 | 0.50 | 0.08 | 0.13 | 0.00 | 0.00 |
|  |  |  | NSV | 6.10 | 0.00 | 0.31 | 0.44 | 0.10 | 0.10 | 0.00 | 0.00 |
| **Red fox** | **2017** | FJ | MNLV | 9.31 | 0.11 | 0.09 | 0.39 | 0.04 | 0.23 | 0.03 | 0.00 |
|  |  |  | NSV | 9.73 | 0.29 | 0.16 | 0.15 | 0.05 | 0.04 | 0.23 | 0.00 |
| **Red fox** | **2017** | LR&I | MNLV | 12.51 | 0.00 | 0.14 | 0.36 | 0.39 | 0.06 | 0.00 | 0.00 |
|  |  |  | NSV | 15.27 | 0.00 | 0.15 | 0.30 | 0.36 | 0.07 | 0.00 | 0.00 |
| **Red fox** | **2019** | S&Br | MNLV | 4.05 | 0.00 | 0.09 | 0.42 | 0.29 | 0.17 | 0.00 | 0.00 |
|  |  |  | NSV | 11.02 | 0.00 | 0.06 | 0.45 | 0.24 | 0.21 | 0.00 | 0.00 |
| **Red fox** | **2018** | W | MNLV | 2.40 | 0.31 | 0.39 | 0.14 | 0.02 | 0.00 | 0.00 | 0.00 |
|  |  |  | NSV | 3.01 | 0.23 | 0.39 | 0.18 | 0.03 | 0.00 | 0.00 | 0.00 |

*Fox individual (or pair) ID

**medians are listed for fox pairs and foxes followed multiple years


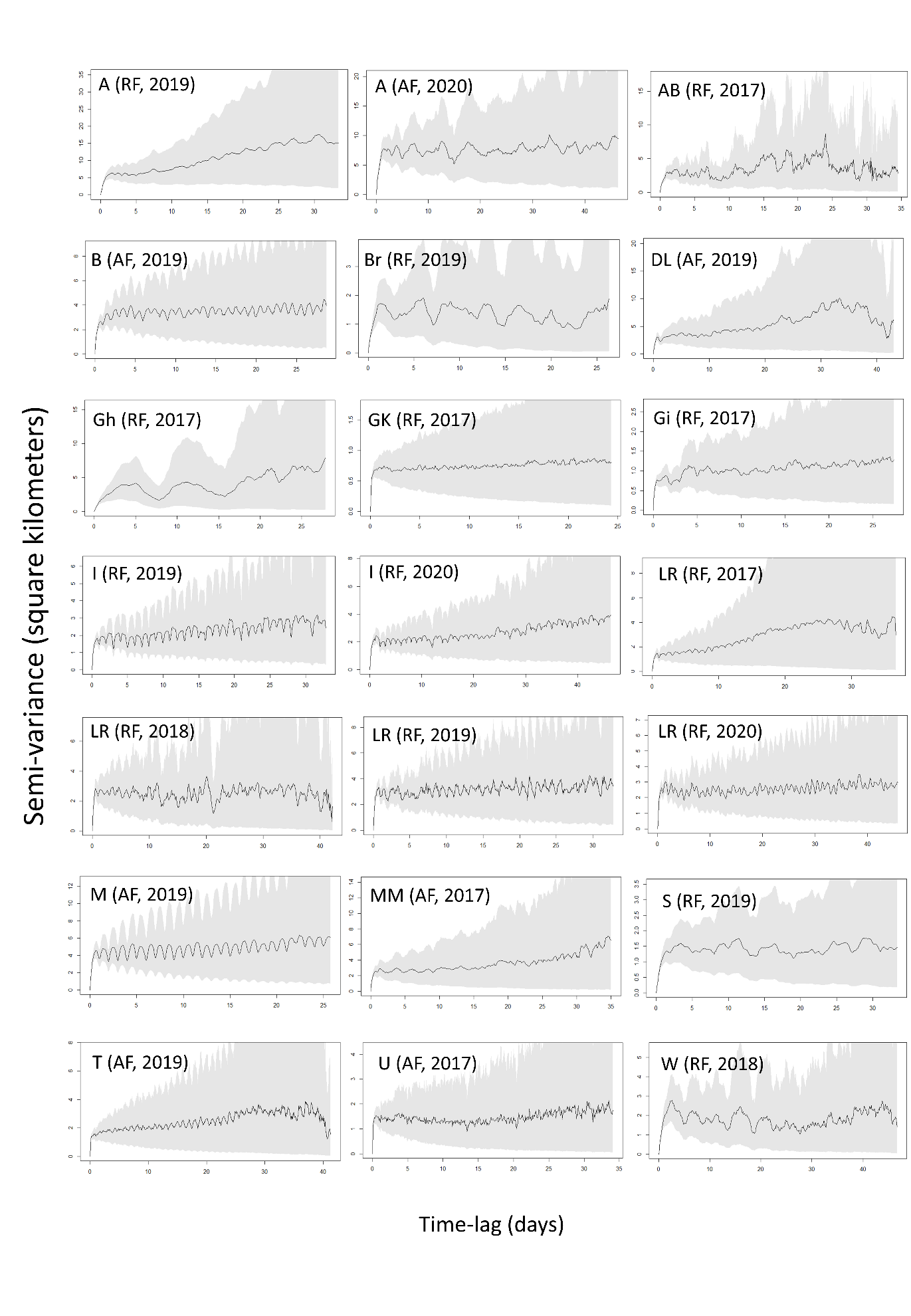


**Fig. S2.** Variograms of foxes identified as resident after inspection of their raw tracks at large time lags (25-40 days). AF = Arctic fox, RF = red fox.


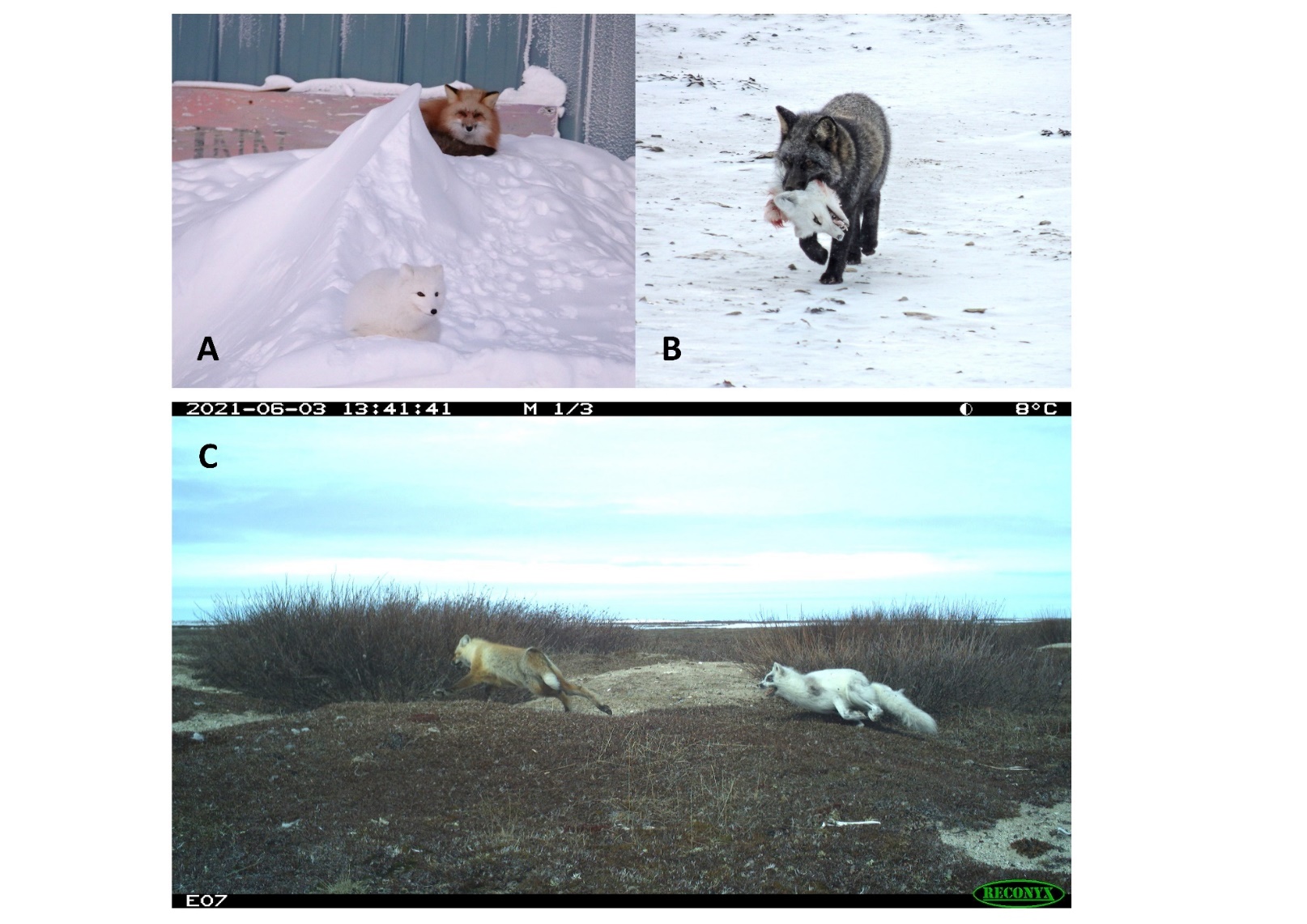


**Fig. S3.** Examples of interactions between Arctic and red foxes in the Churchill and Wapusk area in northeastern Manitoba, Canada. A) The two species can be observed tolerating each other, notably where they may access anthropogenic food subsidies (in town). B) Rarely but regularly, interference interactions can be lethal for the Arctic foxes. These extreme events were always observed in November (B. Debets, pers. obs., November 2014; J. Waterman, pers. obs., November 2017; D. Alcorn, pers. obs., November 2020), which, in Churchill, marks the beginning of food scarcity and harsher climate (Warret Rodrigues and Roth 2023). C) Arctic fox chasing a red fox from its den (interference interaction) in June, when geese have started reproduction and resources are becoming more abundant, but pups are likely born and highly vulnerable (both foxes remained alive at least until the camera stopped working the following week). Photos courtesy of Churchill resident Dave Allcorn (A; March 2022) and Dr. Jane Waterman (B; November 2017), and retrieved from our Reconyx trail camera by Sean Johnson-Bice (C; June 2021).
